# Memristive synapses connect brain and silicon spiking neurons

**DOI:** 10.1038/s41598-020-58831-9

**Published:** 2020-02-25

**Authors:** Alexantrou Serb, Andrea Corna, Richard George, Ali Khiat, Federico Rocchi, Marco Reato, Marta Maschietto, Christian Mayr, Giacomo Indiveri, Stefano Vassanelli, Themistoklis Prodromakis

**Affiliations:** 1 Centre for Electronics Frontiers, University of Southampton, Southampton, SO17 1BJ UK; 2 Biomedical Sciences and Padua Neuroscience Center, University of Padova, Padova, 35131 Italy; 3 Institute of Circuits and Systems, TU Dresden, Dresden, 01062 Germany; 4 Institute of Neuroinformatics, University of Zurich and ETH Zurich, Zurich, 8057 Switzerland

**Keywords:** Bionanoelectronics, Nanosensors

## Abstract

Brain function relies on circuits of spiking neurons with synapses playing the key role of merging transmission with memory storage and processing. Electronics has made important advances to emulate neurons and synapses and brain-computer interfacing concepts that interlink brain and brain-inspired devices are beginning to materialise. We report on memristive links between brain and silicon spiking neurons that emulate transmission and plasticity properties of real synapses. A memristor paired with a metal-thin film titanium oxide microelectrode connects a silicon neuron to a neuron of the rat hippocampus. Memristive plasticity accounts for modulation of connection strength, while transmission is mediated by weighted stimuli through the thin film oxide leading to responses that resemble excitatory postsynaptic potentials. The reverse brain-to-silicon link is established through a microelectrode-memristor pair. On these bases, we demonstrate a three-neuron brain-silicon network where memristive synapses undergo long-term potentiation or depression driven by neuronal firing rates.

## Introduction

Invasive spike-based Brain-Computer Interfaces (BCIs) based on implantable neural interfaces have shown great potential for neural prostheses^1–3^. Currently, spike processing is typically managed by digital Von Neumann-based hardware running statistical algorithms. However, neuromorphic electronic devices and architectures represent a fascinating computational alternative, by virtue of relying on near-biological spike signals and processing strategies^4–6^. In this context, recent findings that nanoscale memristors can emulate plasticity properties of synapses^7,8^ have, on the one hand, boosted hopes of delivering computing systems that are closer to the brain circuits in terms of computation capacity and power efficiency^9,10^. On the other hand, they created the premise for BCIs where spikes are seamlessly processed by nanoscale physical elements, as recently demonstrated for the encoding and sorting of spikes recorded by large-scale multielectrode arrays from neurons in culture^11^. Thus, in perspective, neuroelectronic systems with memristors are promising to ultimately deliver neuromorphic BCIs where silicon and brain neurons are intertwined, sharing signal transmission and processing rules with application in neuroprosthetics^5^ and bioelectronic medicines^12^.

**Figure 1:**
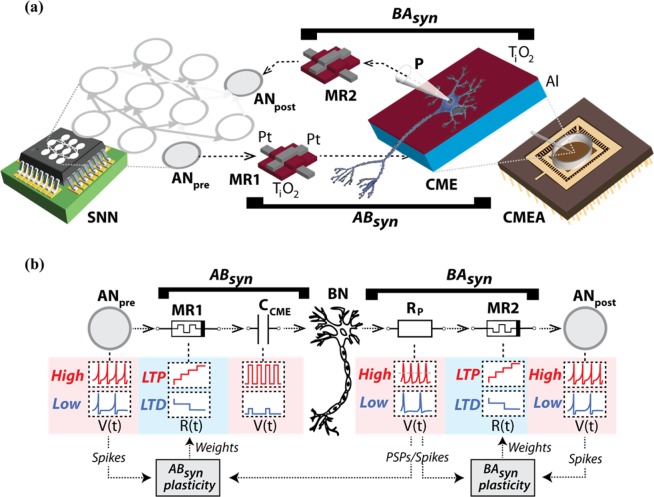
Synaptors connect silicon and brain neurons in hybrid network. (**a**) Sketch of the main components of the hybrid circuit and of the synaptors. *AN*_*pre*_ and AN_post_ are silicon spiking neurons of a VLSI network^28,35^ (SNN), while MR1 and MR2 are Pt/TiO_x_/Pt memristors^36^. The capacitive Al/TiO_2_ electrode, CME, is an element of the multi electrode array, CMEA (Supplementary Fig. 1) where rat hippocampal neurons are cultured on the functionalized surface of the TiO_2_ thin film. One neuron is contacted by a patch-clamp pipette, P, for intracellular whole-cell recording. The two synaptors, *AB*_*syn*_ and *BA*_*syn*_, connect the ‘presynaptic’ silicon neuron (*AN*_*pre*_) to the brain neuron (BN), and BN to the ‘postsynaptic’ silicon neuron, AN_post_. The two memristors, MR1 and MR2, emulate plasticity in the two synaptors, whereas electronics-to-BN and BN-to-electronics signal transmission are mediated by the CME and the patch-clamp electrode. (**b**) Operational scheme. In *AB*_*syn*_, changes in MR1 resistive states, R(t), are driven by *AN*_*pre*_ and BN depolarisations rates according to an approximated BCM plasticity rule (Supplementary Table 1 and Supplementary Fig. 3) resulting in either LTP (red), LTD (blue) or no change. MR1 resistive states are translated into weighted voltage stimuli. These are delivered to BN through the CME capacitance (C_CME_) causing EPSP-like depolarisations, in turn leading to action potential firing (Supplementary Fig. 1). Similarly, in *BA*_*syn*_, BN spikes are recorded by the patch-clamp electrode through its resistance, Rp, threshold-detected and then transmitted to AN_post_ as current injections that are adjusted via MR2 weights.

We hereby demonstrate two memristive connections that link silicon spiking neurons and brain neurons in both directions. The connections emulate synaptic function. In the silicon-to-brain path, a TiO_x_ memristor was coupled to a metal-thin film TiO_2_ microelectrode to connect a very-large-scale-integration (VLSI) spiking neuron to a biological neuron from a rat hippocampus in culture (Fig. 1a). The link, referred to as *artificial-to-biological synaptor* (*AB*_*syn*_), was conceived to emulate both the spike transmission and plasticity processing of a brain synapse. The memristor MR1 stores synaptic weights as resistive states. The thin film capacitive microelectrode^13^ CME delivers stimuli to the biological neuron (BN) that are adjusted by the memristive weights (Fig. 1b). Thus, in analogy with a native synapse, *AB*_*syn*_ operates by injecting in the BN an excitatory current, which reflects a plasticity-dependent synaptic strength. To emulate plasticity, the memristor MR1 is operated as a two-terminal device through a control system that receives pre- and post-synaptic depolarisations from one silicon neuron (*AN*_*pre*_) and one biological neuron (BN), respectively. The plasticity rule is implemented in software, and programming pulses are delivered to change the internal resistance of the device. Resistive states (weights) are translated into adjustable voltage stimuli that, through CME, produce postsynaptic depolarisations in the biological neuron (Fig. 1b and Supplementary Fig. 1). Notably, these capacitively-induced depolarisations resemble native excitatory-postsynaptic potentials (EPSPs), eventually leading to spike firing when the biological cell threshold is exceeded (Supplementary Fig. 1). We conceived the *biological-to-artificial synaptor* (*BA*_*syn*_) following a similar approach. BN spikes are recorded by a patch-clamp microelectrode, then processed by the plasticity-driven memristor MR2, and finally transmitted to a second silicon neuron, AN_post_, via current injection. This configuration comprises a representative example of hybrid circuit connecting silicon spiking neurons to a biological neuron and illustrates how an artificial neuron can influence the firing of another artificial neuron through a biological intermediary without any externally forced signals along the route. In summary, along the forward pathway, the artificial ‘presynaptic’ neuron *AN*_*pre*_ excited BN through *AB*_*syn*_. Through the return branch, BN stimulated the ‘postsynaptic’ silicon neuron AN_post_ through *BA*_*syn*_ (Fig. 1).

An intriguing method for implementing the synaptors involves using the standardised interface of the Internet, which has been previously trialled for non-synaptic network communications^14–16^. We thus instantiated our example of synaptor-linked circuit in a geographically-distributed manner. Three set-ups were connected via user datagram protocol (UDP): a neuromorphic chip hosting silicon spiking neurons (located in Zurich, Switzerland), a memristor handling instrument (Southampton, UK) and a capacitive multi-electrode array with neurons of the rat hippocampus (Padova, Italy) (Supplementary Fig. 2). Notably, the artificial and biological neuron set-ups communicated exclusively via the memristor set-up (see also Supplementary Note 2), thereby univocally establishing the two synaptors. The central position of the memristor set-up within the network rendered it the *de facto* control centre of the entire system.

We report a demonstrative experiment where synaptor plasticity was inspired by the Schaffer collateral - CA1 neuron glutamatergic synapse and the landmark demonstration of Dudek and Bear that high frequency presynaptic activity induces long-term potentiation (LTP) whereas low frequency firing causes long-term depression (LTD)^17^. This behaviour can be interpreted on the basis of the Bienenstock-Cooper-Munro (BCM) theory, modelling the change of synaptic strength as dependent on the product of the input presynaptic activity and a function of the postsynaptic response with a modification threshold accounting for the transition between the two plasticity polarities^17,18^ (Supplementary Fig. 3). For the sake of simplicity, we implemented an approximation of the BCM theory with a constant plasticity modification threshold (i.e. following a Cooper, Liberman and Oja approach^18^), and by splitting plasticity polarities across three frequency ranges (Supplementary Fig. 3 and Supplementary Table 1). During the experiment, the silicon neuron was acting as a pacemaker. Inspired by the Dudek and Bear experimental paradigm, we set *AN*_*pre*_ firing at constant frequencies leading to plasticity changes that were driven by post-synaptic (i.e., BN) activity. In practice, postsynaptic activity was estimated, in terms of depolarisation frequency as measured within a time window immediately preceding each presynaptic spike. Memristor weights were then programmed accordingly (Supplementary Fig. 3b). At the start of the experiment, BN spiking was elicited through *AB*_*syn*_ by setting the silicon neuron *AN*_*pre*_ to fire at high-frequency, eventually leading to LTP of the synaptor. As such, the protocol emulated LTP induction in the Schaffer collateral-CA1 neuron synapse by high frequency discharge of the presynaptic CA3 neuron^17^. It should be noted that following a rate-coded – and not phase-coded– plasticity rule provided a certain degree of immunity against physical and location-dependent internet delays in this experiment, as the specific timing of spikes was secondary in importance to the overall rate.

**Figure 2:**
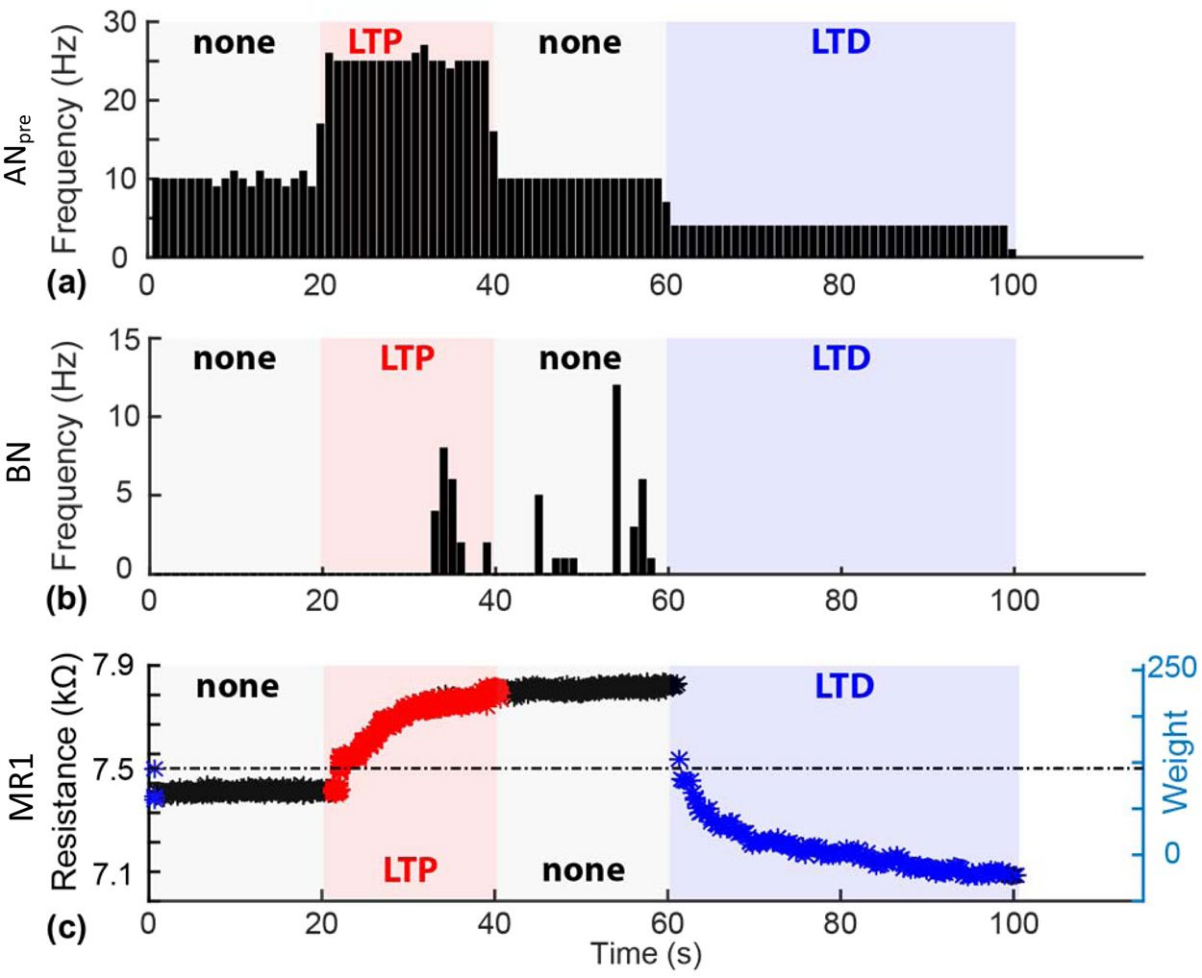
*AB*_*syn*_ plasticity in geographically distributed hybrid circuit. (**a**) Activity pattern of the pacemaker artificial neuron *AN*_*pre*_. Firing frequency is modulated in four phases, targeting the induction of plasticity as per the sequence: none/LTP/none/LTD, using the chosen plasticity rule. (**b**) BN firing response to *AB*_*syn*_ inputs. After LTP induction, the origianl 10 Hz pacemaker stimulation becomes capable of eliciting BN action potentials, thus reflecting the increase of postsynaptic potential amplitudes to above therhold. Firing persists until the commencement of the depotentiation/depression phase. (**c**) MR2 weight evolution. Data points denote resistance values for the intended LTP (red), LTD (blue) or no polarity change (black) phases. The right vertical axis indicates the correpsonding weight. X-axis common to all panels.

Experimental results are summarized in Fig. 2. The pacemaker neuron *AN*_*pre*_ was set to fire regularly at different rates during four subsequent phases of the experiment (i.e., at 10, 25, 10 and 4 Hz, lasting 20, 20, 20 and 40 seconds each). This protocol was designed to cause polarity changes at *AB*_*syn*_ along the pattern ‘none/LTP/none/LTD’ as depicted in Fig. 2a and in accordance with the plasticity rule of Supplementary Table 1. BN spikes recorded by the patch-clamp pipette are shown in Fig. 2b (for amplitudes of subthreshold postsynaptic potentials see Supplementary Fig. 4). Spikes triggered in BN during the high rate 25 Hz discharge of the pacemaker neuron confirmed *AB*_*syn*_ potentiation. Consistent with LTP induction, BN spiking activity persisted during the subsequent phase at 10 Hz presynaptic frequency, thus witnessing no change of plasticity polarity. The subsequent setting of the pacemaker to a low frequency (4 Hz) then caused first depotentiation and eventually LTD of *AB*_*syn*_. The resistance of the *AB*_*syn*_ memristor, MR1, is plotted in Fig. 2 throughout the different phases of the experiment. The evolution of MR1 resistance during the experiment demonstrates the potentiation of synaptor weight (i.e. increase in resistance) during the LTP phase, its maintenance during the ‘none’ phase, and the depotentiation (return of resistance to baseline) and subsequent depression (below starting baseline) during the LTD phase.

**Figure 3:**
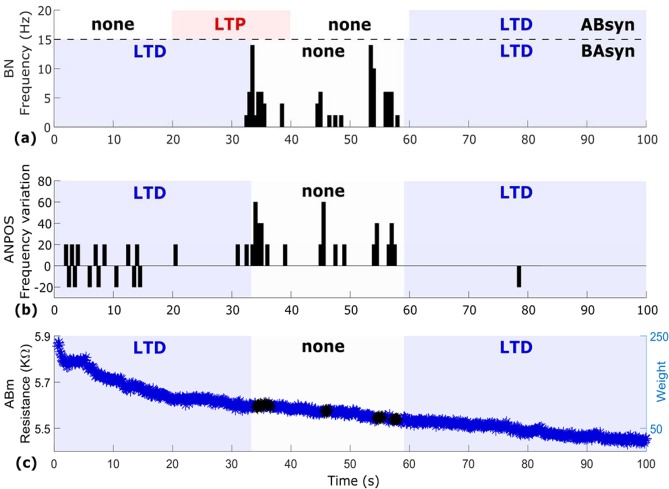
Geographically distributed circuit: return pathway. (**a**) BN firing rate with shadowed areas inidcating plasticity polarity at *AB*_*syn*_ (above the dashed line) and *BA*_*syn*_ (below the deshed line). (**b**) AN_post_ spiking frequency. An increase of spiking activity (expressed as percentage of variation) is observed in the middle of the run in coincidence with BN firing followed by a return to baseline. (**c**) *BA*_*syn*_ weight evolution. The low-rate spiking of BN and AN_post_ caused a strong depression trend (blue) only temporarily reverting to ‘none’ (black) during BN excitation and synchronization of the two neurons. X-axis common to all panels.

Results from the return, biological-to-artificial branch of the circuit –where BN was connected through *BA*_*syn*_ to its post-synaptic target, the silicon neuron AN_post_– are shown in Fig. 3. Importantly, in order to favour plasticity modulation at *BA*_*syn*_, the (artificial) spiking neural network (SNN) environment of AN_post_ (i.e. the artificial neurons-on-chip that AN_post_ connects to) was set to induce a stable background rate of spontaneous firing within AN_post_. Stable AN_post_ spontaneous activity is visible during both LTD phases of *BA*_*syn*_ (Fig. 3b). BN firing, induced by *AB*_*syn*_ potentiation, then triggers additional AN_post_ activity during the ‘no plasticity’ phase of the run, with BN and AN_post_ becoming synchronized (Fig. 3b). The weight evolution of the MR2 memristor (Fig. 3c) is characterised by a dominant depression trend as the low-rate spontaneous activities of BN and AN_post_ hampered LTP induction at *BA*_*syn*_. Only during the brief epochs of sustained BN firing the polarity of plasticity changed (black data points in Fig. 3c), thus favouring temporal summation of high-frequency *BA*_*syn*_ inputs leading to spikes triggering and synchronization of the two neurons.

## Discussion

We have introduced the concept and demonstrated the feasibility of synaptors, two synapse-inspired neuroelectronic links that connect artificial spiking neurons and brain neurons. Two different synaptors, *AB*_*syn*_ and *BA*_*syn*_, respectively enabled the artificial-to-biological and the biological-to-artificial communication by emulating two fundamental functions of the biological synapse: signal transmission and plasticity-mediated signal processing. Our focus here was on demonstrating synaptors with excitatory characteristics.

Synaptors were implemented by relying on two separate physical electronic components: one for signal transmission and one for plasticity. Biological-to-electronic (in *BA*_*syn*_) and electronic-to-biological (in *AB*_*syn*_) signal transmission was realised through microelectrodes. For *BA*_*syn*_, a patch-clamp microelectrode in whole-cell configuration recorded the spikes of the biological neuron. This invasive solution was preferred to non-invasive extracellular microelectrodes as it gave us the opportunity to capture subthreshold responses of BN to *AB*_*syn*_ activity in a very clean manner throughout the reported di-synaptor circuit experiment. Nevertheless, *BA*_*syn*_ is also compatible with extracellular spike recording methods through non-invasive bioelectronic interfaces^19^. Yet, signal transmission represented a severe challenge in the case of *AB*_*syn*_. Along this electronic-to-biological signal pathway, spikes recorded from the artificial neuron had to be transmitted –after appropriate weighting– by eliciting responses similar to EPSPs in the biological neuron. To that end, we deployed a TiO_2_ thin film capacitive microelectrode technology^13,20^ achieving a non-invasive and finely tuneable stimulation. Capacitive stimuli through the thin film and triggered by ‘presynaptic’ spikes caused membrane depolarisations via voltage gated channel opening that resembled EPSPs in terms of temporal dynamics, and that were adjustable in amplitude to match synaptic weight.

TiO_x_ memristors were at the core of plasticity emulation in both synaptors. Plasticity was implemented by pulse programming one memristor (per synapse) to store synaptic weights, which were computed and updated in real-time by a plasticity algorithm based on a BCM-inspired model. *BA*_*syn*_ weights were converted to current injections into the silicon neuron; *AB*_*syn*_ weights, instead, were transformed in depolarising voltage stimuli delivered through the capacitive microelectrode to the biological neuron. Thus, by making an analogy between *AB*_*syn*_ and an excitatory glutamatergic synapse, transmembrane currents induced by capacitive stimulation corresponded to currents through glutamate AMPA receptors; the resistive states of the memristor were changes of AMPA conductance driven by long-term plasticity; the plasticity algorithm was collectively representing the molecular mechanisms leading to changes of AMPA conductance (e.g., NMDA-dependent mechanisms).

The reported di-synaptor circuit showcased the functionality of the two types of synaptor within a plasticity-driven hybrid circuit. Conceptually, the circuit demonstrates an elementary BCI formed by a neuromorphic architecture of spiking neurons that seamlessly interact with the brain though synapse-inspired communication pathways. Our demonstration leads to two observations that merit discussion. First, our chosen experimental set-up was that of a feedforward chain of three neurons communicating over long distance. The chain is controlled by a single signal input: the forced firing of the neuron at the start of the chain (*AN*_*pre*_). At this point, we note that in contrast to fully electronic brain-inspired systems, biology introduces nondeterministic components that render network behaviour difficult to predict analytically. This raises the challenges of first being able to describe the function of such hybrid systems and then developing reliable benchmarking strategies. In our case, this phenomenon manifests itself as a pattern of well-controlled plasticity phases at the forward path synapse and then a less directly controlled pattern of plasticity induction (of the form LTD/none/LTD) at the backward path synapse, as shown in Fig. 3c. This behaviour, however, is reproduced during the repeat (validation) run, as shown in Supplementary Fig. 7, thus showing that at least some consistency of results can be expected (and also validating that the concept and its underlying hardware/software infrastructure operate correctly).

Secondly, the experiment shows successful synaptor operation over the internet and not only by wire connection. Crucially, synaptors can be understood as geographically distributed synapses, with different components of the synapse physically located in separate places (e.g. the weight is stored in a memristor and the executive arm of the synapse is located at the capacitive/current-injection interface). Achieving this is not trivial, since issues such as handling UDP propagation delays (which are typically variable and thus difficult to control) need to be resolved. To that end, we employed a technique of referencing secondary partner spikes to the primary partner (see methods section) and used rate-dependent plasticity. The referencing technique effectively makes a remote memristor set-up appear and operate as if it were sitting next to the secondary partner, thus making the whole synaptor appear as if it were located in a single place. This also implies that if communication from primary to secondary partner is one-way, internet network delays can be de-facto eliminated completely from the operation of the biohybrid network. For reference, UDP timing measurements indicate variable static delays from 10–90 ms across European connections, with the timing of individual UDP packets along a connection varying below 2 ms, i.e. the relative timing of pulses is stable. However, completely compensating for round-trip delays cannot be achieved using this technique (closed loop systems will have to be able to tolerate round-trip delays). Nevertheless, synaptors represent the first example of a geographically distributed hybrid network of artificial and biological neurons connected through physical synapse-like elements. Intriguingly, whereas brain evolution has had to face tight physical constraints that spatially confined connectivity, synaptors are suitable for overcoming such barriers and enabling mixed biological/brain-inspired architectures that are globally interconnected (from small groups of sub-neural networks hosted on a few PCs around the world to potentially a huge web of IoT-interconnected devices).

In perspective, synaptors are suitable for improvements with respect to both signal transmission and plasticity emulation. Nanoscale electrodes^21–24^ can enhance the quality of interfacing and provide selectivity for neuronal compartments, and *in vivo* interfaces extend the use of synaptors to BCIs in the living animal^5^. Developments in the field of low-voltage operated memristive devices^25^ will help reducing the burden of signal amplification, while analogue emulation of plasticity and the extension to phase-dependent plasticity rules (e.g. STDP)^26,27^ will further expand the application potential for smart bioelectronic medicines and BCIs. For example, synaptors may be employed for adaptive bioelectronic control of autonomous reflexes (e.g. for therapy of heart arrhythmias, hypertension or bladder dysfunction by neurostimulation of the peripheral nervous system) or for therapy and rehabilitation in neurological patients (e.g. in spinal cord injury or Parkinson’s).

## Methods

### Silicon spiking neurons and AER-based communication

The central part of the artificial side of the bio-hybrid system is formed by a reconfigurable on-line learning spiking neuromorphic processor (ROLLS)^28^, which contains neuromorphic CMOS circuits emulating short-term plasticity (STP) properties of synapses^29^ and long-term plasticity (LTP) ones^30^. In addition, this processor comprises mixed signal analogue-digital circuits which implement a model of the adaptive exponential integrate-and-fire neuron^31^. Input and output spikes are sent/transmitted from the chip using asynchronous IO logic circuits which employ the Address-Event-Representation (AER) communication protocol^32^. The chip is connected to a host PC which receives UDP-packets from the internet. These packets contain information on stimulus destinations and corresponding synaptic weights. This information is decoded by a Field Programmable Gate Array (FPGA) device and conveyed to the neuromorphic processor. In this work, the parameters of the CMOS synapse circuits were set to produce weak excitatory postsynaptic currents (EPSCs) with long time constants, such that high frequency stimulation causes an additive effect on the net amplitude of the resulting EPSC. The value of the weight encoded in the UDP packet was used to produce spike trains of different frequencies transmitted by the FPGA to the neuromorphic processor (see also Supplementary Fig. 4). In addition to the signals arriving from the UDP interface, locally generated spike trains were sent to the neuromorphic processor, to provide a controlled stimulus for evoking background activity. This system is shown in Supplementary Fig. 4.

### Memristors

The memristive synapse set-up consisted of an array of memristive devices positioned inside an ArC memristor characterisation and testing instrument^33^ (Supplementary Fig. 5. http:www.arc-instruments.co.uk). The instrument is controlled by a PC, which handles all the communications over UDP; all through a python-based user interface. The software is configured to react to UDP packets carrying information about the firing of either artificial or biological neurons (who fired when). Once a packet is received, the ID of the neuron that emitted it and the time of spiking are both retrieved from the packet payload and the neural connectivity matrix (held at the Southampton set-up) is consulted in order to determine which neurons are pre- and which are post-synaptic to the firing cell. Then, if the plasticity conditions are met, the ArC instrument applies programming pulses that cause the memristive synapses to change their resistive states. Importantly, the set-up can control whether LTP- or LTD-type plasticity is to be applied in each case, but once the pulses have been applied it is the device responses that determine the magnitude of the plasticity. Notably, resistivity transitions of the device are non-volatile, they hold over at least hours^27^ as also exemplified in our prototype experiment and are therefore fully compatible with typical LTP and LTD time scales of natural synapses. The system is sustained by a specific methodology for handling timing within the overall network (Zurich, Southampton, Padova). The set-up in Southampton being the node that links Zurich and Padova together, controls the overall handling of time. Under this system, one of the partners (in our case Zurich) is labelled as the’primary partner’ and all timing information arriving from that partner is treated as a ground truth. Every timing information sent by other partners then has to be related to this ground truth, for example if the primary partner says that neuron 12 fires a spike at time 305, then the secondary partner(s) is informed of this (through Southampton). If then a neuron in the secondary partner set-up fires 5 time units (as measured by a wall-clock) after being informed of the firing of neuron 12, it emits a packet informing Southampton that e.g. neuron 55 fired at time 310. This way the relative timing between spikes arriving from the primary partner and the spikes triggered by the secondary partner(s) in response is maintained despite any network delays. The price is that if the secondary partners wish to communicate spikes to the primary partner, network delays for the entire round-trip are then burdening the secondary-to-primary pathway. The details of timing control at each partner site are fairly complicated and constrained by the set-ups at each partner, but all timing information is eventually encoded in an’absolute time’ record held at Southampton. The rationale behind this design decision was to ensure that at least in the pathway from primary to secondary partner(s) timing control is sufficiently tight to sustain plasticity in the face of network delays.

### Neuronal culture and electrophysiology

Embryonic (E18) rat hippocampal neurons were plated and cultured on the CMEA according to procedures described in detail in^34^. Recordings were performed on 8–12 DIV neurons. The experimental setup in UNIPD (Supplementary Fig. 1) enabled UDP-triggered capacitive stimulation of neurons^13^ while simultaneously recording and communicating via UDP the occurrence of depolarisations that were measured by patch-clamp whole-cell recording. The CMEA (20 × 20 independent TiO_2_ capacitors, each one of area 50 × 50 µm^2^) was controlled by a dedicated stimulation board and all the connections to partners, Southampton and Zurich, were managed by a PC running a LabVIEW-based software (National Instruments Corp, Austin, TX, USA). The stimulation protocol was derived from^13^ and further optimized for non-invasive adjustable stimulation of the neurons. In brief, capacitive stimulation was adjusted to the memristor’s resistance (i.e. the synaptor weight) by varying the repetition number of appropriate stimulation waveforms (Supplementary Fig. 1).Patch-Clamp recordings were performed in whole-cell current-clamp configuration using an Axopatch 200B amplifier (Molecular Devices, USA) connected to the PC through a BNC-2110 Shielded Connector Block (National Instruments Corp, Austin, TX, USA) along with a PCI-6259 PCI Card (National Instruments Corp, Austin, TX, USA). WinWCP (Strathclyde Electrophysiology Software, University of Strathclyde, Glasgow, UK) was used for data acquisition. Micropipettes were pulled from borosilicate glass capillaries (GB150T-10, Science Products GmbH, Hofheim, Germany) using a P-97 Flaming/Brown Micropipette Puller (Sutter Instruments Corp., Novato, CA, USA). Intracellular pipette solution and extracellular solution used during the experiments were respectively (in mM): 6.0 KCl, 120 K gluconate, 10 HEPES, 3.0 EGTA, 5 MgATP, 20 Sucrose (adjusted to pH 7.3 with 1N KOH); 135.0 NaCl, 5.4 KCl, 1.0 MgCl2, 1.8 CaCl2, 10.0 Glucose, 5.0 HEPES (adjusted to pH 7.4 with 1N NaOH). Digitised recordings were analysed by a custom LabVIEW software running on the PC, allowing detection and discrimination of firing and EPSP activity through a thresholding approach.

All experiments were performed in accordance with the Italian and European legislation for the use of animals for scientific purposes and protocols approved by the ethical committee of the University of Padova and by the Italian Ministry of Health (authorisation number 522/2018-PR).

## Supplementary information

Supplementary information
