# Author Correction: Memristive synapses connect brain and silicon spiking neurons

**DOI:** 10.1038/s41598-020-66548-y

**Published:** 2020-06-09

**Authors:** Alexantrou Serb, Andrea Corna, Richard George, Ali Khiat, Federico Rocchi, Marco Reato, Marta Maschietto, Christian Mayr, Giacomo Indiveri, Stefano Vassanelli, Themistoklis Prodromakis

**Affiliations:** 1 Centre for Electronics Frontiers, University of Southampton, Southampton, SO17 1BJ UK; 2 Biomedical Sciences and Padua Neuroscience Center, University of Padova, Padova, 35131 Italy; 3 Institute of Circuits and Systems, TU Dresden, Dresden, 01062 Germany; 4 Institute of Neuroinformatics, University of Zurich and ETH Zurich, Zurich, 8057 Switzerland

Correction to: *Scientific Reports* 10.1038/s41598-020-58831-9, published online 25 February 2020

The Acknowledgements section in this Article was omitted. The Acknowledgements section should read:

“The authors would like to acknowledge financial support from the European Commission, Future and Emerging Technology programme, through the projects RAMP (6112058) and SYNCH (824162) and from the Engineering and Physical Sciences Engineering Research Council programme FORTE (EP/R024642/1). All data are available at: 10.5258/SOTON/D1280”

