# Supplementary material for: Memristive synapses connect brain and silicon spiking neurons
Source: Sci Rep. 2020 Feb 25;10:2590. doi: 10.1038/s41598-020-58831-9 (PMC7042282; doi:10.1038/s41598-020-58831-9)
Supplement: Supplementary file 1 — Supplementary information [file 41598_2020_58831_MOESM1_ESM.pdf]

SUPPLEMENTARY MATERIAL

Memristive synapses connect brain and silicon spiking neurons

Alexantrou Serb[1], Andrea Corna[2], Richard George[3], Ali Khiat[1], Federico Rocchi[2], Marco Reato[2], Marta Maschietto[2], Christian Mayr[3], Giacomo Indiveri[4], Stefano Vassanelli[2], Themistoklis Prodromakis[1,*]

[1]Electronic Materials & Devices Research Group, Zepler Institute for Photonics & Nanoelectronics, University of Southampton, SO17 1BJ, UK

[2]Biomedical Sciences and Padua Neuroscience Center, University of Padova, 35131, Italy

[3]Institute of Circuits and Systems, TU Dresden, 01062, Germany

[4]Institute of Neuroinformatics, University of Zurich and ETH Zurich, 8057, Switzerland

[*]Corresponding Authors: Prof Themis Prodromakis (Email: t.prodromakis@soton.ac.uk) or Prof Stefano Vassanelli (Email: stefano.vassanelli@unipd.it)

Supplementary figures and tables

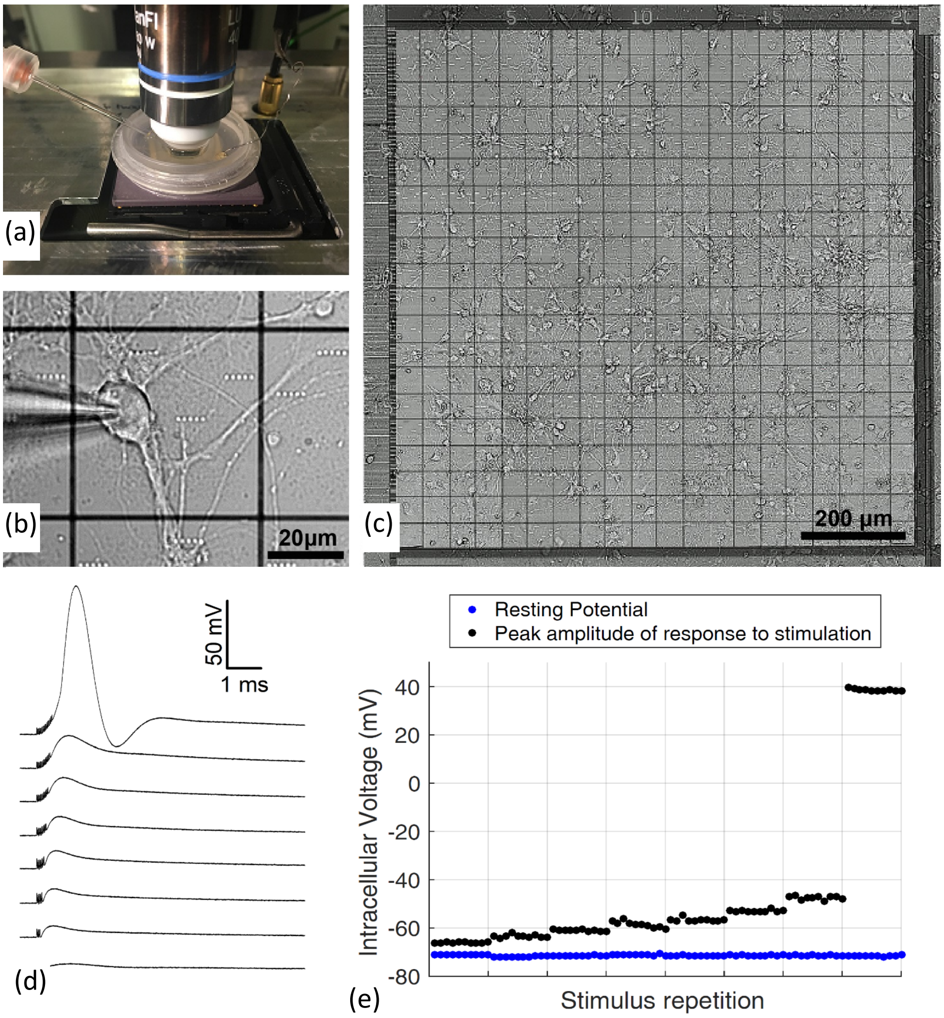

Supplementary figure 1 | BN stimulation and recording setup. (a) CMEA chip mounted on a stimulation board. A Perspex chamber on the chip contains the extracellular recording solution. The patch pipette is on the left. (b) Micrograph of BN on the capacitive microelectrode (square at the canter of the image) and recorded through the patch-clamp pipette in whole-cell configuration. (c) 10 DIV network of hippocampal neurons in culture on the 20 x 20 array of capacitive microelectrodes. (d) Examples of whole cell patch clamp recordings showing BN depolarisations caused by capacitive stimulations of increasing intensity (from bottom to top). Stimulations artefacts caused by voltage pulse repetitions (2 to 16, from bottom to top) are followed by EPSP-like depolarisations of increasing amplitude, until a suprathreshold depolarization is accompanied by action potential firing (top trace). (e) Peak amplitude (black dots) of neuronal depolarisations induced by the different stimuli reported in d (ten repetitions at 1 s interval). Blue dots indicate the intracellular potential measured immediately before stimulus application and therefore identifying the resting potential of the neuron at the time of stimulation.

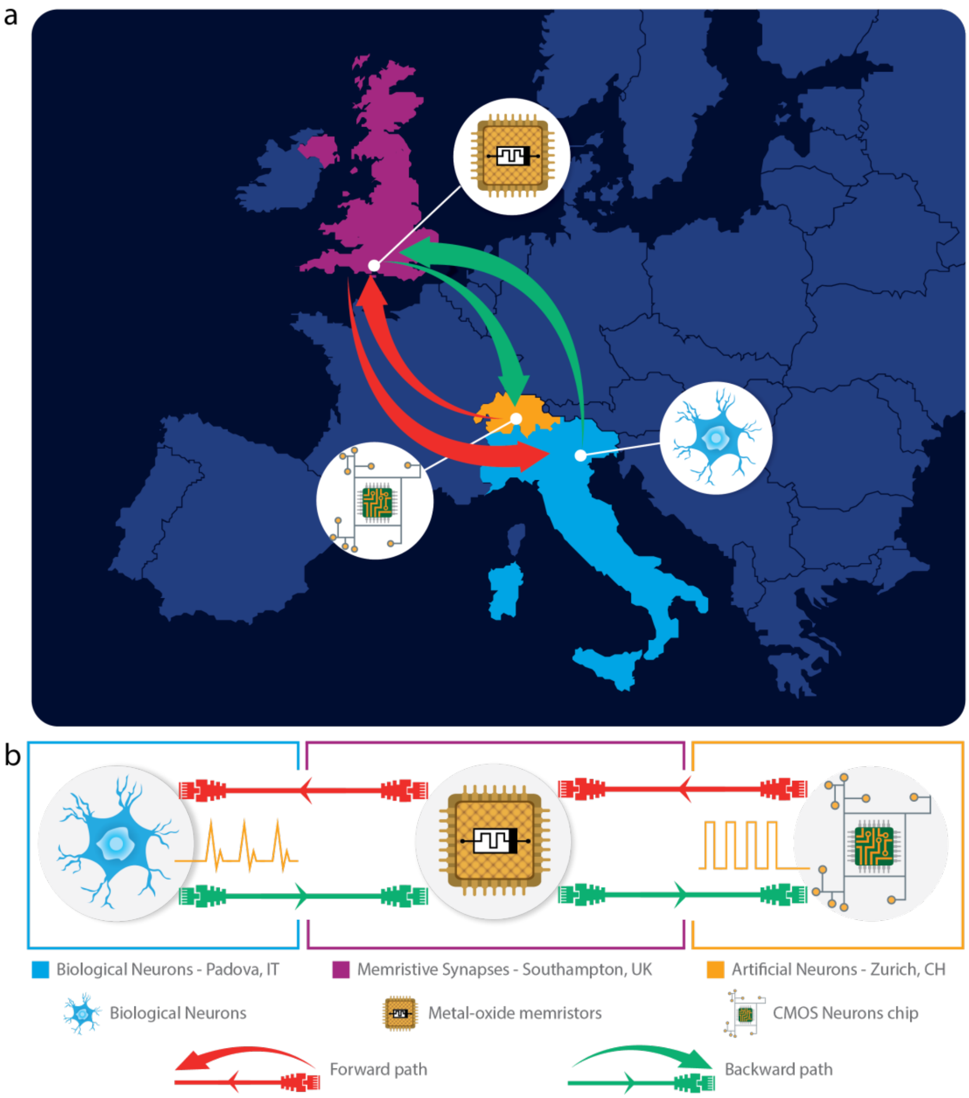

Supplementary figure 2 | Geographically distributed network. (a) Geographical location of artificial/biological neurons and memristors: biological neurons in Padova (IT), artificial neurons in Zurich (CH) and memristors in Southampton (UK). Forward and reverse signal pathways are illustrated by red and green arrows respectively. (b) High-level connectivity diagram.

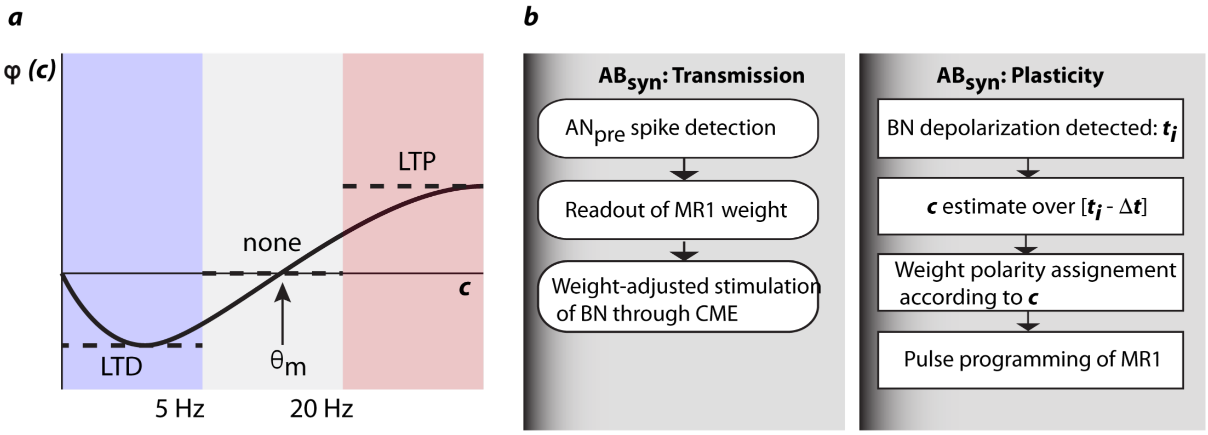

Supplementary figure 3 | Synaptor's plasticity. ***a*. Approximation of the BCM rule used to program MR1 and MR2 in the synaptors. In the original BCM theory and its Cooper–Lieberman–Oja (CLO) precursor[18], the change of synaptic strength, *m*, for a given presynaptic frequency, *d*, is proportional to a function $\varphi(c)$ of the postsynaptic frequency *c* according to the relation: $dm/dt = \varphi(c)d$ (black line). θ_m is the modification threshold –which is constant in the CLO theory–, i.e. the value of postsynaptic activity at which plasticity changes polarity. In our approximation of the model, plasticity polarity is assigned as indicated by dashed lines and summarised in supplementary table 1, with θ_m fixed across the whole interval [5,20] Hz. *b*. Flowchart of the algorithm for adjusting AB_{syn} strength by pulse programming of MR1 according to the approximated plasticity model in *a*. MR1 was programmed by applying either a positive or a negative pulse for the two plasticity polarities, i.e. potentiation (LTP) and depression (LTD), respectively. Noteworthy, memristance changes induced by pulses were subject to intrinsic variability, recalling the variability of EPSP amplitudes of biological synapses, including the Shaffer collateral-CA1 synapse[39]. MR2 plasticity, in BA_{syn}, was implemented following a similar scheme (no shown)**

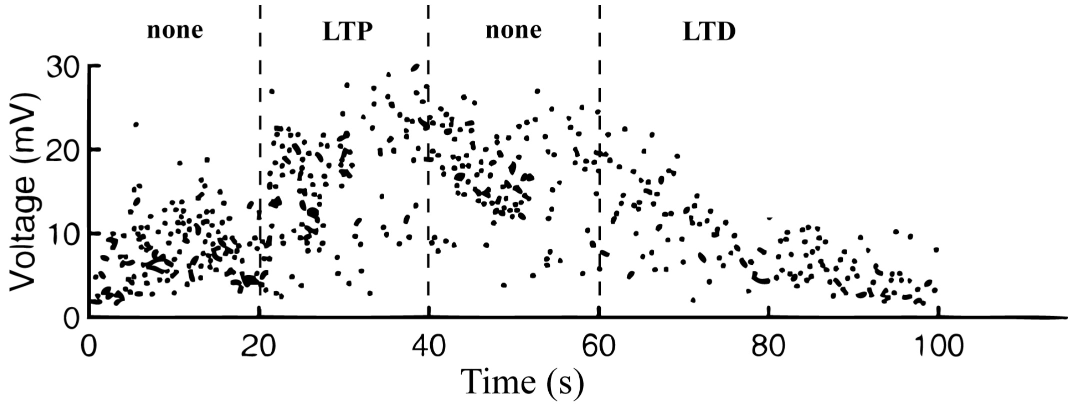

Supplementary figure 4 | Peak amplitudes of subthreshold postsynaptic depolarisations recorded in BN in response to AN_{pre} spikes during the experiment reported in figure 2. Postsynaptic potentials amplitudes were changing in accordance with MR1 plasticity phases (none, LTP, none, LTD).

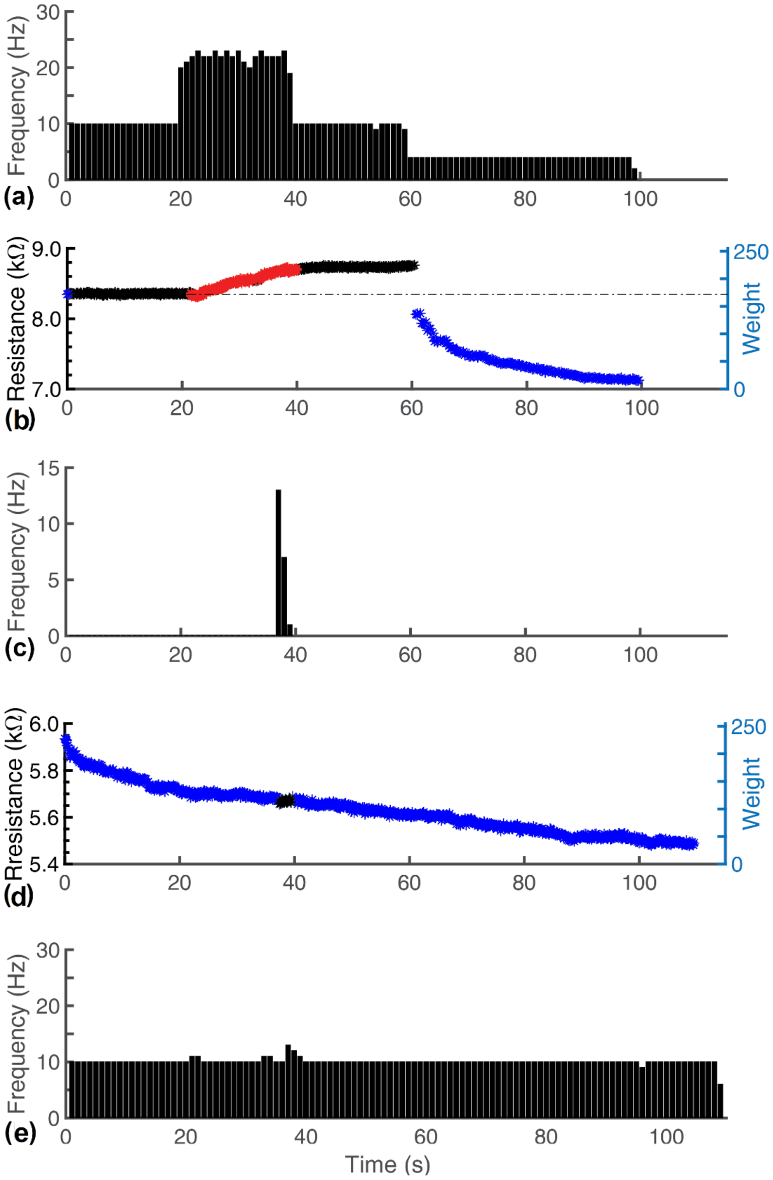

Supplementary figure 5: repetition of the experiment shown in Figures 2 and 3. (a) AN_{pre} activity pattern. (b) AN_{pre} to BN plasticity evolution (memristor MR1). (c) BN firing. (d) BN to AN_{post} plasticity evolution (memristor MR2). (e) ANP_{post} activity pattern. Differences in firing rates with respect to the experiment of figures 2 and 3 are due to different initial synaptors weights, their evolutions and BN responses. However, the overall response remained qualitatively the same, proving both reliability and flexibility of the system to complex dynamics.

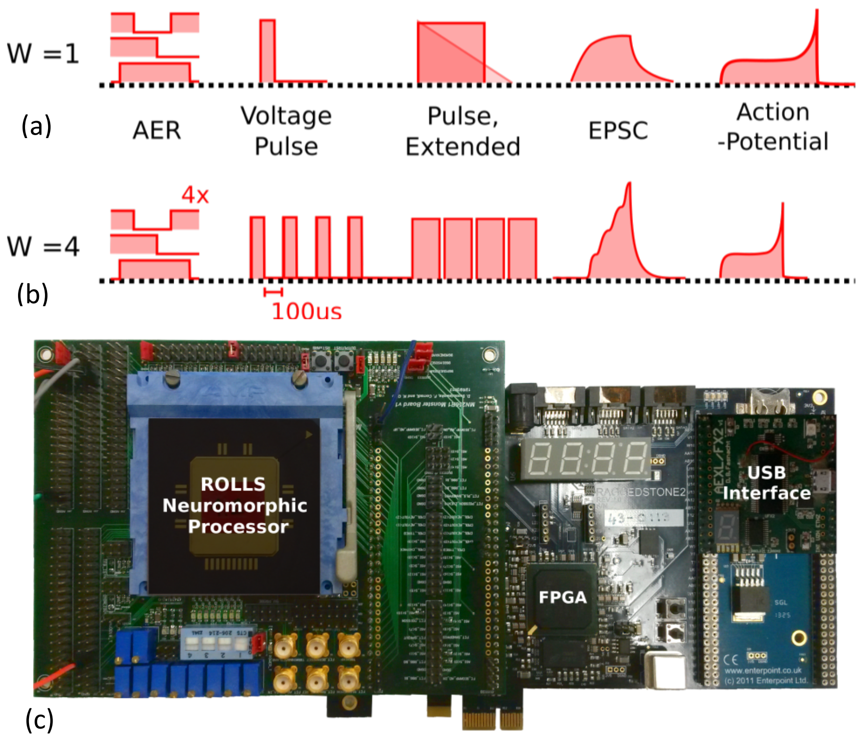

Supplementary figure 6: Artificial neuron set-up, located in Zurich, Switzerland. (a,b) Two examples of how an incoming UDP packet is handled in the cases where the input weight is equal to 1 – i.e. minimum weight quantum – (a) and 4 respectively (b). Note the difference in the EPSC waveforms generated as a result. (c) Artificial neuron set-up located in Zurich, Switzerland.

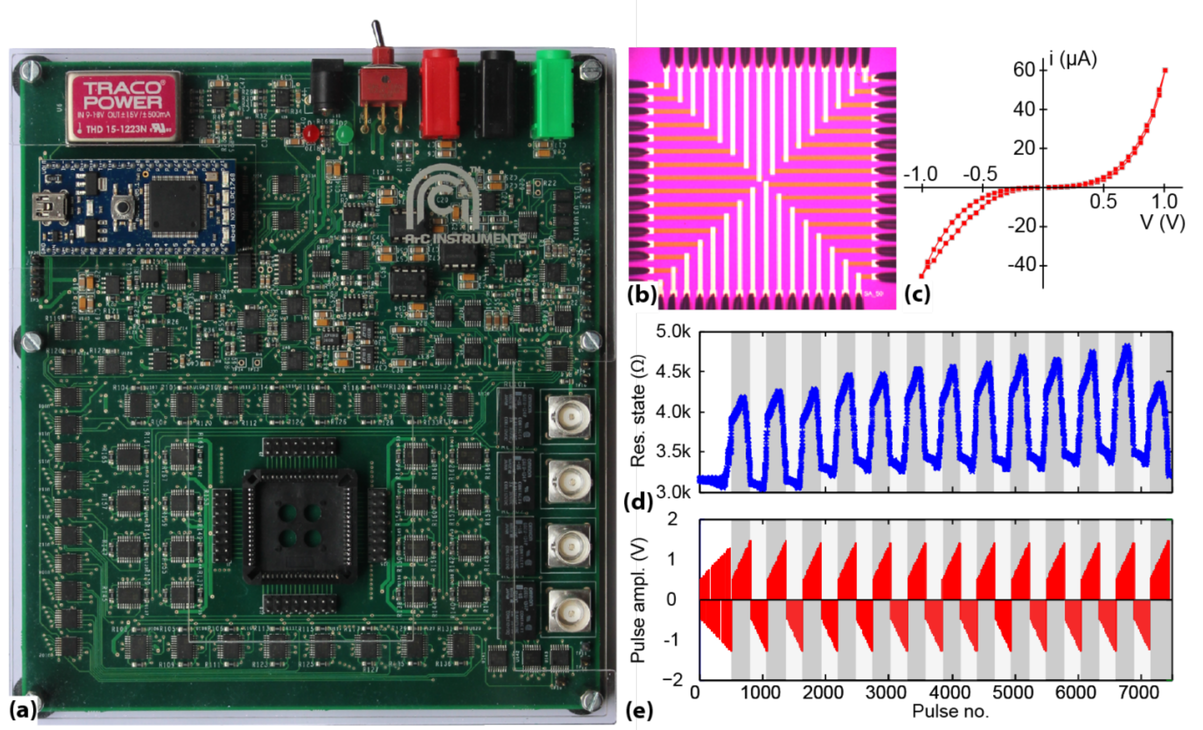

Supplementary figure 7: Memristive synapse set-up core located in Southampton, UK. (a) ArC board. The devices sit within the PLCC68 package socket featuring prominently at the lower middle of the board. (b) Example of stand-alone device array (microphotograph). (c) Typical current-voltage (i-V) characteristic of metal-oxide AlOx-TiOx bilayer devices used in this work. A small amount of pinched hysteresis is observable in this deliberately low voltage/low invasiveness i-V run. (d) Device resistive state modulation in response to stimulation in (e).

R1	Neuron ID	R2	Timestamp
8 bits	24 bits	8 bits	24 bits

Supplementary figure 8: UDP packet structure used in this work. The packet consists of 64 bits in total, broken into four segments as indicated by the labels. Labels correspond to standard use of packets in AER protocol with segments R1 and R2 corresponding to flexible space to be used by various applications utilising the AER packet format. The specific uses of each segment in this work are summarised in Supplementary table 1.

Supplementary tables

Supplementary table 1 | BCM approximation for plasticity polarity

AN_{post} firing rate range (Hz)	Plasticity polarity
<5	LTD
[5, 20]	None
>20	LTP

	R1	Neuron ID	R2	Timestamp
**Padova (secondary)**	Partner identifier (primary/secondary)	Spiking neuron ID	Type of event (PSP, forced AP, spontaneous AP)	Absolute time
**Southampton (synapse)**	Partner identifier (primary/secondary)	Postsynaptic neuron ID	Weight	Absolute time
**Zurich (primary)**	Partner identifier (primary/secondary)	Spiking neuron ID	-nothing-	General relative time

Supplementary table 2: UDP packet payloads by packet segment and partner. Cell entries refer to the type of packet produced by each partner (not the type of packet received by each partner).

Supplementary notes

Supplementary note 1: UDP packet structure and contents.

The UDP packets used in this work follow the structure shown in Supplementary Figure 7 and carry a total of four variable values as payload. The meanings of these variables for each partner are summarised in Supplementary Table 1. For all partners, segment R1 contains a partner identifier value specifying whether the packet is arriving from a primary, synapse or secondary partner. The neuron ID segment contains the identity of the neuron firing if this is emitted by a partner hosting neurons (primary/secondary). This is all the information required by the synapse partner in order to compute which post-synaptic neurons need to be stimulated through the memristor-based synapses. As a result, the synapse partner sends the ID of the postsynaptic neurons to be stimulated. Note that whist the primary and secondary partners send a packet per neuron firing, the synapse partner then emits a packet for each stimulated synapse in response. Segment R2 is used by the biological neuron partner to inform the synapse partner on whether the event that is being communicated in each packet is a PSP, an action potential (AP) resulting

from stimulation or a spontaneous AP. The synapse partner uses the same segment to communicate the strength of the stimulation corresponding to each stimulated synapse, in effect the weight of the synapse. Finally, the timestamp segment communicates timing information using different protocols for each partner. Timing management details are covered in Supplementary note 2.

Supplementary note 2: Timing protocol summary.

The synapse set-up, being the node that links biological and artificial partners together, controls the overall handling of time during operation. Under this system, one of the partners (in this case Zurich, which hosts the neuron that starts the signal path - ANPRE) is labelled as the 'primary partner' and all timing information arriving from that partner is treated as ground truth. Spiking activity from the secondary partner (or in the case of larger systems: partners) is then referenced against this forced ground truth. For example, if the primary partner asserts that neuron X fires a spike at time t_0, then the secondary partner is informed of this, and responds in the form of a post-synaptic potential (PSP) evoked at t_0 through the synapse set-up. If then a neuron Y in the secondary partner set-up fires Δt (wall-clock) time units after being informed of the firing of neuron X, it emits a packet informing the synapse set-up that e.g. neuron Y fired at time $t_0 + \Delta t$. This way the relative timing between spikes arriving from the primary partner and the spikes triggered in response, by the secondary partner, is maintained despite any network delays. In exchange, when the secondary partner wishes to communicate spikes to the primary partner, all network delays for the entire round-trip will burden that communication. Our design ensures that at least in the pathway from primary to secondary partner, timing control is sufficiently tight to sustain timing-sensitive plasticity.

Each partner in the set-up handles time differently: The primary partner operates in 'general relative time', whereby UDP packets inform the synapse set-up of the identity of the neuron currently spiking and the time interval between the present spike and the previous spike, regardless of the origin of the previous spike. Therefore, if neuron 1 spikes at time 12000 and neuron 2 spikes at time 12012, the UDP packet will contain the information: ID=1, dt=12. The synapse set-up operates on the basis of absolute time, i.e. it constructs a sequence of events on a constantly advancing time axis and translates general relative timestamps arriving from the primary partner into absolute time. It then informs all partners of the spiking of every other partner in absolute time. Finally, the secondary partner operates on the basis of a wall-clock that is reset every time information on a primary partner spike arrives (relative time). The secondary partner communicates its own spikes back to the synapse set-up in absolute time.
